# Layered Trichalcogenidophosphate: A New Catalyst Family for Water Splitting

**DOI:** 10.1007/s40820-018-0220-6

**Published:** 2018-08-30

**Authors:** Cheng-Feng Du, Qinghua Liang, Raksha Dangol, Jin Zhao, Hao Ren, Srinivasan Madhavi, Qingyu Yan

**Affiliations:** 1 State Key Laboratory of Solidification Processing, Center of Advanced Lubrication and Seal Materials, Northwestern Polytechnical University, Xi’an, 710072 Shaanxi People’s Republic of China; 2 School of Materials Science and Engineering, Nanyang Technological University, 50 Nanyang Avenue, Singapore, 639798 Singapore

**Keywords:** Two-dimensional materials, Trichalcogenidophosphate, Photocatalysis, Electrocatalysis, Water splitting

## Abstract

Due to the rapidly increasing demand for energy and environmental sustainability, stable and economical hydrogen production has received increasing attention in the past decades. In this regard, hydrogen production through photo- or electrocatalytic water splitting has continued to gain ever-growing interest. However, the existing catalysts are still unable to fulfill the demands of high-efficiency, low-cost, and sustainable hydrogen production. Layered metal trichalcogenidophosphate (MPQ_3_) is a newly developed two-dimensional material with tunable composition and electronic structure. Recently, MPQ_3_ has been considered a promising candidate for clean energy generation and related water splitting applications. In this minireview, we firstly introduce the structure and methods for the synthesis of MPQ_3_ materials. In the following sections, recent developments of MPQ_3_ materials for photo- and electrocatalytic water splitting are briefly summarized. The roles of MPQ_3_ materials in different reaction systems are also discussed. Finally, the challenges related to and prospects of MPQ_3_ materials are presented on the basis of the current developments.
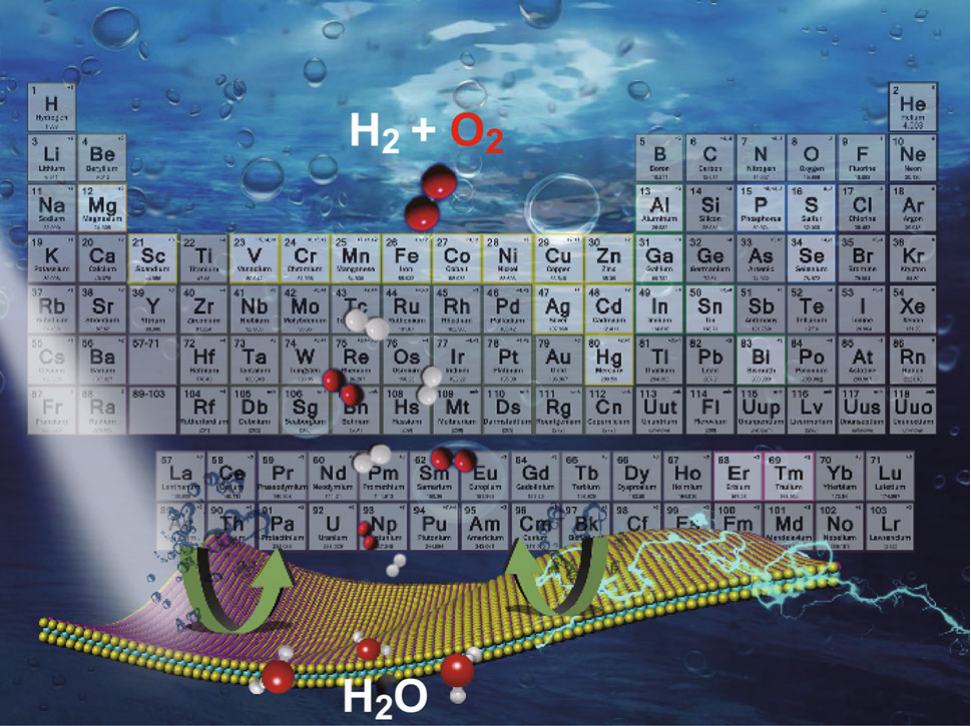

## Highlights

- Layered metal trichalcogenidophosphate (MPQ_3_) is a newly developed 2D material with tunable composition and electronic structure, and is a promising candidate for clean energy generation and related water splitting applications.
- There are no comprehensive studies on layered MPQ_3_ materials for photo- and electrocatalytic water splitting; here, we provide a brief summary of recent work and offer an overview of this promising field.

## Introduction

With the rapidly increasing demand for energy and extensive use of fossil fuels, there is an urgent need to find alternative sources of clean energy to solve the ever-growing environmental problems. Hydrogen, the simplest and most abundant element in the universe, has a high energy density and is sustainable and eco-friendly. The combustion of hydrogen with air/oxygen offers high energy and gives off water as the end product. Hence, hydrogen is considered one of the most promising candidates for fulfilling future energy demands [1, 2]. It has been long recognized that water can be decomposed under solar excitation in plants. Electrical power can also be used to facilitate water splitting into gaseous hydrogen and oxygen, which can be stored for further use. However, it is still a huge challenge to split water on the industrial scale as efficiently as in plants. Moreover, the state-of-the-art electrocatalytic techniques for water splitting still rely heavily on noble metal catalysts such as Pt for the hydrogen evolution reaction (HER) and IrO_2_ for the oxygen evolution reaction (OER). Thus, the generation of hydrogen and oxygen is a high-cost operation, making the use of hydrogen and oxygen as commercial fuels challenging. Therefore, it is highly desirable to find earth-abundant, cost-effective, and highly active catalysts for water splitting [3].

As requirements of effective photocatalytic water splitting, the catalysts should possess a suitable bandgap and carrier mobility [4]. The photo-generated excitons (commonly known as electron–hole pairs) must migrate separately to the surface of the catalyst and be consumed by corresponding redox reactions. In order to prevent recombination of the excited electron and hole, the redox reactions should proceed within the lifetime of these species [4–7]. In this regard, band structure engineering and charge carrier concentration tuning are highly important. Further, as key factors, the catalyst must enable fast charge transfer kinetics and lower the energy for chemisorption of various intermediate species on the catalyst surface to improve the electrochemical water splitting efficiency [8–11]. It has been demonstrated that the chemisorption energies of intermediate species on the catalyst surface can be adjusted by varying the chemical composition of the electrocatalysts [10]. For instance, phosphorus-doped pyrite-type cobalt disulfide exhibits excellent hydrogen evolution activity because of effective reduction in the energy for adsorption of atomic hydrogen on the adjacent cobalt sites doped with phosphorus atoms [11–13]. Therefore, in order to meet the above-mentioned requirements, variation of the composition of the catalyst should be effective for adjusting the bandgap and electronic structures, charge carrier concentration and mobility modulation, and regulation of the chemisorption energy in semiconductor-based catalytic systems.

Layered metal trichalcogenidophosphates, known as metal phosphorus trichalcogenides, have attracted increasing attention in recent years. Since the first discovery of metal chalcogenidophosphate by Friedel more than a century ago [14], a large family of these layered compounds has been synthesized and studied. The general formula for these compounds can be expressed as M^II^PQ_3_ or M_0.5_^I^M_0.5_^III^PQ_3_, where M^II^ represents bivalent metals such as Mg, V, Mn, Fe, Co, Ni, Zn, Cd, Sn, and Hg; M^I^ represents metal ions such as Cu and Ag; and M^III^ can be Cr, V, Al, Ga, In, Bi, Sc, Er, or Tm; Q is a chalcogen, i.e., S or Se (to be more concise, the abbreviation MPQ_3_ is used hereinafter) [14–47]. The diversity of metal, phosphorus, and chalcogen atoms in MPQ_3_ materials offers vast possibilities for achieving the desired physical, chemical, optical, and electronic properties. For instance, the presence of sulfur and phosphorous in metal trithiophosphate compounds exerts a synergistic effect on the surface electronic structure of the central metal atoms [48, 49]. Moreover, the asymmetric M^I^ and M^III^ cations possess different metal–chalcogen bond distances, resulting in differences in the valence band maximum (VBM) and conduction band minimum (CBM) orbital distribution [49]. Moreover, due to the layered structure of MPQ_3_ materials, they can be easily prepared as two-dimensional nanostructures having a large surface area and numerous exposed active sites. Due to these merits, MPQ_3_ materials are widely considered good candidates as high-performance photo- or electrocatalytic water splitting catalysts.

Although there have been several reviews involving layered MPQ_3_ in recent years, there is still no specific summary on layered MPQ_3_ materials. In addition, it is important to summarize the progress on the MPQ_3_ materials used for photo- and electrocatalytic water splitting due to the recent rapid development. In this regard, we aim to provide a brief summary of recent work and offer an overview of this promising field. In this minireview, we provide a brief introduction of the structure of MPQ_3_ materials and subsequently discuss the methods of synthesis, highlighting the advantages and limitations. In the following sections, recent developments of MPQ_3_ materials for photo- and electrocatalytic water splitting are briefly summarized. The roles of MPQ_3_ materials in different reaction systems are also discussed. Finally, the challenges and prospects of MPQ_3_ materials are proposed on the basis of their current developments.

## Crystal Structure and Synthesis of Layered MPQ_3_

**Fig. 1:**
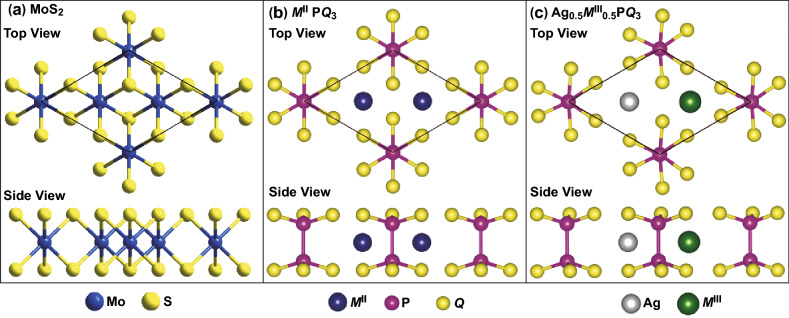
Crystal structures of **a** MoS_2_, **b** M^II^PQ_3_ (M^II^ = Zn, Cd, Mg; X = S, Se), and **c** Ag_0.5_M_0.5_^III^PQ_3_ (M^III^ = Sc, In; Q = S, Se). Bonds between metal atoms and Q atoms are not shown in **b** and **c** [49]. Reproduced with permission. Copyright (2014), AIP Publishing

Generally, trithio- and triseleno-phosphate compounds are considered to have a layered crystal structure similar to that of transition metal disulfides (TMDs, e.g., MoS_2_). Figure 1 shows the typical crystal structures of MoS_2_, M^II^PQ_3_, and Ag_0.5_M_0.5_^III^PQ_3_. In contrast to the structure of MoS_2_, P–P pairs substitute one third of the metal atoms within the MPQ_3_ layer. Therefore, each MQ_6_ octahedron is surrounded by three P_2_Q_6_ groups. The metal layer in MPQ_3_ is encapsulated by both chalcogens and phosphorus atoms. In the case of Ag_0.5_M_0.5_^III^PQ_3_ particularly, the asymmetric Ag^I^ and M^III^ cations possess different metal–chalcogen binding distances, resulting in a distorted P_2_Q_6_ polyhedron (Fig. 1c, Ag_0.5_M_0.5_^III^PQ_3_) [49].

Conventionally, high-quality MPQ_3_ single crystals are synthesized by the chemical vapor transport (CVT) method, in which pure elemental powders are used as the starting materials and a small amount of iodine is used as the transport agent. These starting materials are vacuum-sealed in a quartz tube, which is then placed in a horizontal tube furnace with a temperature gradient. During the CVT process, the transport agent carries the starting materials from the hot end to the cold end of the tube for crystal growth. The crystal growth temperature is usually higher than 600 °C and may vary depending on the metal species. The growth period ranges from several days to even 2 months [24]. Finally, in order to obtain the nanosheets (i.e., single- or few-layered MPQ_3_ products), exfoliation techniques such as micromechanical exfoliation or liquid exfoliation are carried out [43, 50, 51]. Limited by the high reaction temperature, long growth period, and possible explosion hazards, the CVT method is unsuitable for large-scale production of MPQ_3_ materials.

Compared with the CVT method, chemical vapor deposition (CVD) is more suitable for the large-scale growth of MPQ_3_. In a modified CVD method for the synthesis of NiPS_3_ [3], elemental sulfur and phosphorus are evaporated in the two-zoned tube furnace under Ar flow; Ni(OH)_2_ nanosheets grown on various substrates as a metal source are simultaneously heated in another zone. The reaction time for this CVD process is only 1 h, which is greatly reduced when compared with the CVT method. Due to the non-exclusive reaction environment and the reduced reaction time, the CVD process is considered a safer and faster method for producing MPQ_3_ nanosheets. However, the requirement for a highly pure inert atmosphere and complex reaction conditions may limit scalable production.

Recently, a method termed solid-state transformation (SST) was developed for the large-scale production of MPQ_3_ materials [4]. In a typical SST process, metal hydroxides are chosen as starting materials, and elemental sulfur and phosphorus are directly ground with the hydroxides. The mixture is vacuum-sealed in a Pyrex or quartz tube and heated at a certain temperature. After removing the excess sulfur and phosphorus, the MPQ_3_ materials can be obtained. Depending on the compositions and morphologies of the starting metal hydroxides, the products can be either nanosheets or other nanostructures. The reaction temperature of the SST method may be even lower than that of the CVT and CVD methods. With such a low reaction temperature and no requirement for the two-zone furnace, the SST method is regarded as a promising technique for mass production of MPQ_3_.

## MPQ_3_ for Photocatalytic Water Splitting

As one of the most promising approaches to solving the world’s energy and environmental issues, semiconductor-based photocatalytic water splitting has gained a great deal of attention since its first discovery in the last century [52]. The water splitting reactions that occur on the photocatalyst can be simply summarized as follows [7, 53, 54]:

1 $$2{\text{H}}^{ + } + 2{\text{e}}^{ - } \to {\text{H}}_{2}$$

2 $$2{\text{H}}_{2} {\text{O}} + 4{\text{h}}^{ + } \to 4{\text{H}}^{ + } + {\text{O}}_{2}$$

where e^−^ and h^+^ are the photo-generated electron and hole. In order to realize overall water splitting, the semiconductor must have a more negative CBM than the H^+^/H_2_ energy level, and the VBM should be more positive than the O_2_/H_2_O energy level. Therefore, the standard Gibbs free energy (Δ*G*) of at least 1.23 eV needs to be overcome [7, 49]. In addition, the wavelength of ultraviolet and visible light for photocatalysis ranges from 200 to 780 nm. Therefore, the bandgap of the semiconductor-based photocatalysts should be in the range of 1.59–6.20 eV.

**Fig. 2:**
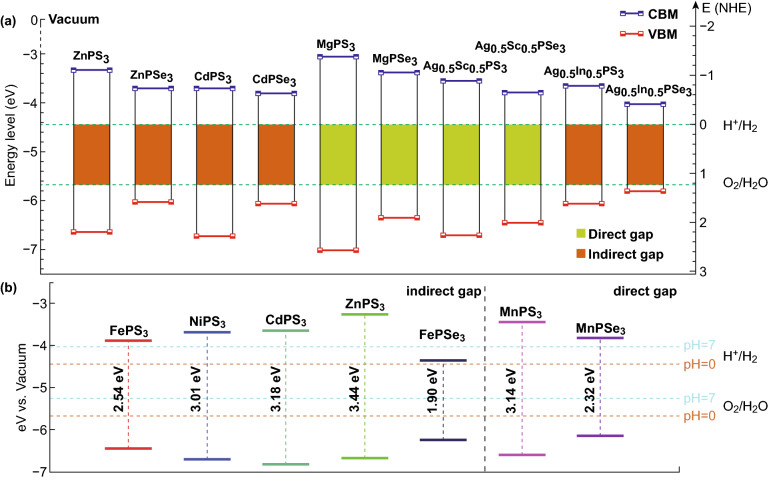
**a** Band-edge positions of MPQ_3_ single layer. The energy scale is indicated by either the normal hydrogen electrode (NHE) (right *Y*-axis) or the vacuum level (left *Y*-axis) in electron volts as a reference. The redox potentials (green dashed line) for water splitting are shown for comparison [49]. Copyright (2001), Macmillan Publishers Ltd. **b** Location of VBM and CBM, calculated with HSE06 functional, for MPS_3_ and MPSe_3_ single layers. The redox potentials for water splitting at pH = 0 (orange dashed lines) and pH = 7 (cyan dashed lines) are shown for comparison [55]. Reproduced with permission. Copyright (2016), Wiley–VCH, GmbH & Co. KGaA. (Color figure online)

The bandgap structures of many single-layered group II trichalcogenidophosphates (M^II^PQ_3_, M^II^ = Mg, Zn, Cd) and group III silver trichalcogenidophosphates (Ag_0.5_M_0.5_^III^PQ_3_, M^III^ = Sc, In) have been studied based on density functional theory (DFT) calculations [49]. As shown in Fig. 2a, Liu and Peng aligned the CBM and VBM of these compounds with respect to the water redox potential levels and evaluated their redox abilities. In consideration of the stability of single-layered MPQ_3_ and the changes in the redox potential based on the pH, Zhou and co-workers also studied the band edges and the optical properties of MPS_3_ (M = Mn, Fe, Ni, Zn, Cd) and MPSe_3_ (M = Mn, Fe) using theoretical calculations [55]. Using the theoretically calculated optical absorption coefficient (*α*) of a series of single-layer MPQ_3_ species, MnPSe_3_, FePQ_3_, and NiPS_3_ were found to exhibit optical absorption in the visible spectral range (1.59–3.26 eV) [55]. A similar tendency was also experimentally observed by Kloc and Xiong [50]. Notably, among the studied compounds, FePSe_3_ was found to exhibit the narrowest bandgap based on both calculated and experimental data. However, FePSe_3_ was not suitable for water splitting at pH = 7 as the CBM is lower than the reduction potential of H^+^/H_2_, whereas MnPSe_3_ showed strong adsorption in the visible spectral region. Further, the calculated carrier mobility of single-layered MnPSe_3_ is much higher than that of many other 2D materials (electron mobility: 625.9 cm^2^ V^−1^ S^−1^; hole mobility: 34.7 cm^2^ V^−1^ S^−1^). The huge difference between the mobility of these two carriers suggests effective separation of the photo-generated electron–hole pairs during the photocatalytic process. This makes MnPSe_3_ a promising candidate for high-efficiency photocatalytic water splitting.

**Fig. 3:**
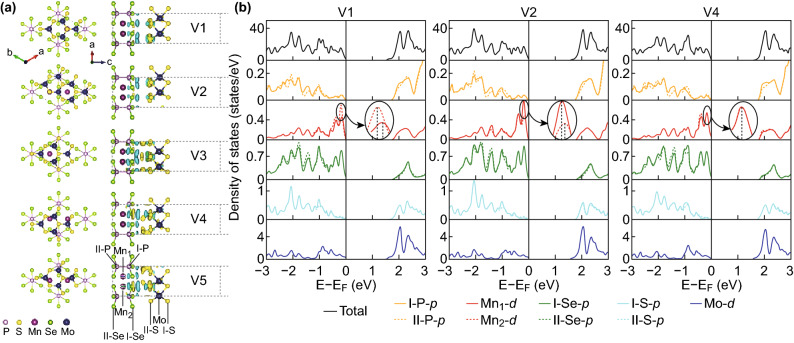
**a** Structure and side view of the charge density difference of MnPSe_3_/MoS_2_ heterostructures with different stacking models V1–V5. The isosurface value is 0.15 e nm^−3^. Yellow (blue) regions represent the net charge gain (loss). **b** Total and partial DOS of V1, V2, and V4 configurations. Fermi level is indicated by the vertical shadow line and set to zero [56]. Reproduced with permission. Copyright (2017), Macmillan Publishers Ltd. (Color figure online)

Apart from the individual materials, the combination of photoactive materials with other co-catalysts to form an integrated system is another important field in photocatalytic research. Therefore, it is necessary to study the electronic structure of the heterojunctions in these integrated systems. Mi and co-workers recently explored the electronic structure of MnPSe_3_-based 2D van der Waals heterostructures using DFT calculations. In the case of the MnPSe_3_/MoS_2_ heterostructure [56], different stacking patterns of single-layered MnPSe_3_ and MoS_2_ were studied, as presented in Fig. 3a. In some stacking patterns, spin splitting at the VBM of the MnPSe_3_ layer was observed due to hybridization of the d orbital in Mn, thus enhancing the electron mobility (Fig. 3b). In addition, MnPSe_3_/MoS_2_ is a type II heterostructure in which the top part of the valence band is mainly contributed by MnPSe_3_, while the bottom part of the conduction band is mainly contributed by MoS_2_. Typically, the type I heterostructure has a symmetrical offset of potential barriers for the electrons and holes, where direct exciton transition occurs at the heterointerface. In type II heterostructures, the electrons and holes are localized on different sides of the heterointerface, which results in an indirect exciton transition [57–59]. The type II band alignment results in separation of the photo-generated electrons and holes, thus enhancing the photocatalytic efficiency. They also calculated the electronic structure of MnPSe_3_/CrSiTe_3_, where MnPSe_3_ and CrSiTe_3_ possess similar crystal structures and have a low lattice mismatch (about 4.7%) [60]. In this work, Mi et al. studied the effects of strain and an electric field on the heterostructures. When the heterostructure was formed under tensile strain, the band-edge position shifted from type I to type II, accompanied by a transition from an indirect bandgap to direct bandgap. When a compressive strain was applied, the heterostructure changed from semiconducting to conducting. At the same time, the band alignment could be tuned to type I or type II by applying a suitable electric field. The two mentioned studies demonstrated the possibility of band structure modulation of the MnPSe_3_-based van der Waals heterostructure, suggesting the potential applicability of the novel heterostructures in photo-electronics.

Although MnPSe_3_ has gained extensive attention and shows great potential in the field of photocatalytic water splitting, experimental studies are still focused on the fundamental physical properties [50, 61]. Recently, He and co-workers turned their attention to Fe- and Ni-based compounds instead of Mn-based trichalcogenidophosphates [62, 63]. They conducted an experimental study of few-layered nickel trithiophosphate (NiPS_3_) prepared by a modified CVD process for photocatalytic hydrogen evolution [62]. The resultant NiPS_3_ nanosheets had a lateral size larger than 15 μm and thickness of less than 3.5 nm. The as-synthesized ultrathin NiPS_3_ nanosheets could be used for photo-hydrogen evolution in neutral and pure water under xenon light or simulated AM1.5G solar illumination with a constant evolution rate of ~ 26.42 μmol g^−1^ h^−1^. However, the intrinsic driving force was not energetically sufficient for oxygen evolution due to the misaligned valance band of NiPS_3_ relative to the water oxidation potential. Therefore, the NiPS_3_ nanosheets suffered from a disadvantage similar to that of many other single-component photocatalysts, wherein the photoactivity is degraded by photo-anodic corrosion. This is because during the photocatalytic process, the photo-generated electrons are consumed by H_2_ generation, while the photo-generated holes accumulate due to the misaligned valance band of NiPS_3_ relative to the water oxidation potential. The highly oxidizing photo-generated holes then react with NiPS_3_ itself [53]. Consequently, this study experimentally confirms the photocatalytic activity of NiPS_3_. Nevertheless, the performance of NiPS_3_ is much poorer than that of the commercially available materials, and this material is still far from practical application [7, 53]. Further study toward the design and construction of novel MPQ_3_-based photocatalysts is still needed.

**Fig. 4:**
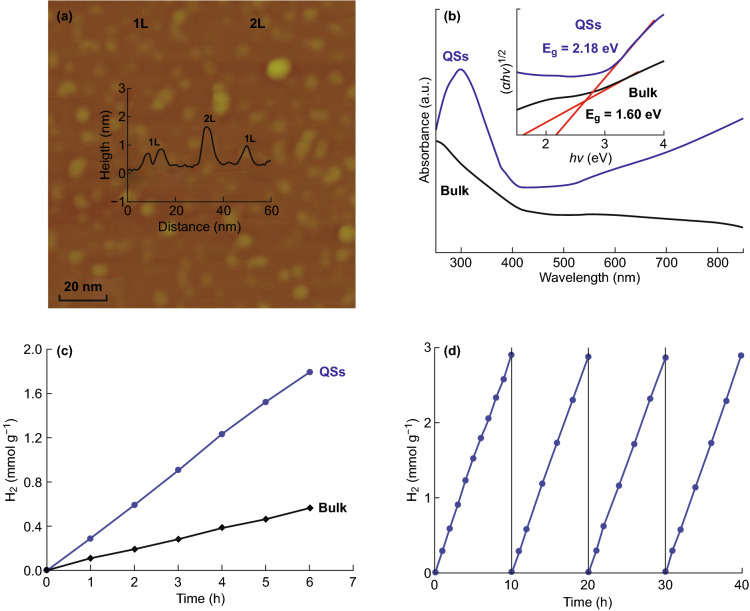
**a** AFM images and corresponding height analysis of FePS_3_ quantum sheets. **b** UV–Vis/NIR diffuse reflectance absorption spectra and estimated bandgap potential (inset) of synthesized FePS_3_ bulk (1.60 eV) and quantum sheets (2.18 eV). **c** Plots of hydrogen production through direct photocatalytic water splitting of the FePS_3_ bulk and quantum sheets in 100 mL deionized water with 10% TEOA as sacrificial agent. **d** Cycling measurements of hydrogen gas evolution with FePS_3_ quantum sheets [63]. Reproduced with permission. Copyright (2018), Wiley–VCH, GmbH & Co. KGaA

The photocatalytic hydrogen evolution performance of FePS_3_ compounds was also recently studied [63]. He and co-workers developed a two-step hydrazine intercalation and exfoliation process for the synthesis of single-layered FePS_3_ quantum sheets from bulk FePS_3_ crystals. The resulting quantum sheets possessed a lateral size of 4–8 nm and thickness of less than 2 nm (Fig. 4a). As discussed above, the bandgap is closely related to the photocatalytic activity. In the case of the FePS_3_ quantum sheets, the decreased size from bulk to quantum sheets increased the bandgap (Fig. 4b). Therefore, the hydrogen production efficiency of the FePS_3_ quantum sheets was three times higher (290 μmol g^−1^ h^−1^) than that of the bulk FePS_3_ (Fig. 4c). This value is around 11-fold higher than that of the NiPS_3_ nanosheets [62], but still about an order of magnitude lower than that of the MoS_2_-based materials [64] and Ni/Cu-modified titania [65]. Furthermore, measurement of the hydrogen evolution over 40 h indicated that these FePS_3_ quantum sheets were more stable than the NiPS_3_ nanosheets (Fig. 4d). Given that the reduced size of the quantum sheets leads to the exposure of numerous boundaries and active sites, the authors attributed the enhanced performance to the effective separation of photo-excited electrons and holes. When FePS_3_ and NiPS_3_ are compared with other MPQ_3_ compounds, the relatively narrower bandgap of FePS_3_ and NiPS_3_ is thought to be the main contributor to their visible light photocatalytic activity. However, the narrow bandgap also results in insufficient exciton energy. Therefore, dopant metals such as Mn, Zn, and Cd that can broaden the bandgap might be helpful for bandgap engineering and heterostructure construction.

## MPQ_3_ for Electrocatalytic Hydrogen Evolution

Photocatalytic water splitting relies strongly on the absorption of solar energy, making this technique generally inefficient. In contrast, electrochemical catalysis, as one of the fundamental processes in electrochemistry, offers a more feasible and practical option [1]. In the past several decades, researchers have paid more attention to non-noble metal-based electrocatalysts due to their low cost. As a kind of semiconductor with tunable and suitable physiochemical properties, layered MPQ_3_ compounds were also evaluated for electrocatalytic hydrogen evolution. A general mechanism for the electrochemical HER process might include an adsorption step and desorption steps as follows [66–68]:

Volmer adsorption step:

3 $${\text{In}}\;{\text{acidic}}\;{\text{solution:}}\;{\text{H}}_{3} {\text{O}}^{ + } + {\text{e}}^{ - } + {\text{Cat}} \to {\text{Cat}} - {\text{H}} + {\text{H}}_{2} {\text{O}}$$

4 $${\text{In}}\;{\text{alkaline}}\;{\text{solution:}}\;{\text{H}}_{2} {\text{O}} + {\text{e}}^{ - } + {\text{Cat}} \to {\text{Cat}} - {\text{H}} + {\text{OH}}^{ - }$$

Heyrovsky desorption step:

5 $${\text{In}}\;{\text{acidic}}\;{\text{solution:}}\;{\text{Cat}} - {\text{H}} + {\text{H}}_{3} {\text{O}}^{ + } + {\text{e}}^{ - } \to {\text{H}}_{2} + {\text{H}}_{2} {\text{O}} + {\text{Cat}}$$

6 $${\text{In}}\;{\text{alkaline}}\;{\text{solution:}}\;{\text{Cat}} - {\text{H}} + {\text{H}}_{2} {\text{O}} + {\text{e}}^{ - } \to {\text{H}}_{2} + {\text{OH}}^{ - } + {\text{Cat}}$$

Tafel desorption step:

7 $$2{\text{Cat}} - {\text{H}} \to {\text{H}}_{2} + 2{\text{Cat}}$$

where Cat is the catalyst and Cat − H refers to the adsorbed H atom on the catalyst surface. Depending on the desorption step, the whole HER process can follow the Volmer–Heyrovsky or Volmer–Tafel pathway. Because the HER is the cathodic half reaction of electrochemical water splitting, the self-corrosion (such as photo-anodic corrosion) that occurs during photocatalytic water splitting could be avoided.

Recently, Sampath and co-workers reported the HER performance of liquid-exfoliated NiPS_3_ nanosheets over a wide pH range of 1–14 and in a neutral 3.5 wt% NaCl solution that simulates seawater [67]. The bulk NiPS_3_ crystals were firstly synthesized through the CVT method. After exfoliation in the liquid phase, NiPS_3_ nanosheets with a lateral size of 200–400 nm and thickness of 0.65–0.7 nm were obtained. The exfoliated NiPS_3_ nanosheets showed improved electrochemical HER performance under all the pH conditions. The overpotential of the exfoliated NiPS_3_ nanosheets at 10 mA cm^−2^ in pH = 1, 14, and neutral 3.5 wt% NaCl solution was 297, 398, and 816 mV, respectively. The authors also studied the HER activity through DFT calculation. By evaluating the free energy of hydrogen adsorption (Δ*G*_H_), they concluded that the phosphorus atom is the preferred site for H adsorption, consistent with the inference from metal phosphides [69, 70]. Later, Sampath’s group reported a similar study on the HER performance of exfoliated FePS_3_ nanosheets [71]. The FePS_3_ nanosheets demonstrated better HER performance than the Ni-based analogs, where the overpotential at 10 mA cm^−2^ in pH = 1, 14, and neutral 3.5 wt% NaCl solution was 211 ± 3, 337 ± 4, and 637 ± 4 mV, respectively. When combined with reduced graphene oxide (rGO), the HER performance of both compounds could be further improved. The authors ascribed this enhancement to the improved conductivity derived from rGO.

Pumera et al. [68] also reported a systematic study of the electrocatalytic HER performance under acidic conditions for a series of MPS_3_ compounds, including MnPS_3_, FePS_3_, CoPS_3_, NiPS_3_, ZnPS_3_, CdPS_3_, and SnPS_3_. The overpotentials of NiPS_3_ and CoPS_3_ were 530 and 590 mV at a current density of 10 mA cm^−2^, respectively. Here, the overpotential of FePS_3_ (860 mV) was found to be higher than that of its Ni and Co analogs. The overpotentials of the other MPS_3_ compounds were all higher than that of the bare glassy carbon electrode. According to their results, it is interesting that bulk NiPS_3_ had a lower overpotential than bulk FePS_3_, whereas Sampath et al. reported that exfoliated NiPS_3_ nanosheets possess a higher overpotential than exfoliated FePS_3_ nanosheets. Therefore, further study still needs to be conducted to explore the intrinsic properties of these materials.

**Fig. 5:**
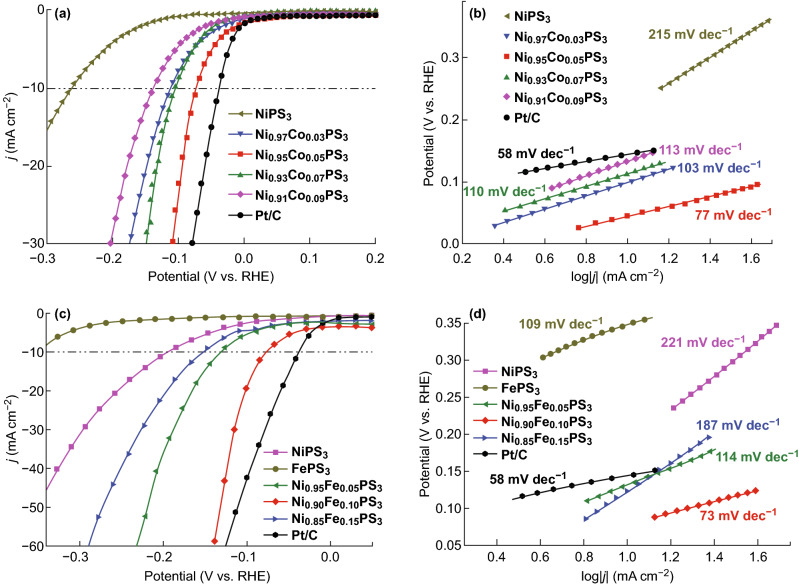
Electrochemical properties of as-prepared Ni_1−*x*_Co_*x*_PS_3_ and Ni_1−*x*_Fe_*x*_PS_3_ nanosheets for the HER in 1 m KOH. **a** *J*–*V* curves after *i*R correction for application of NiPS_3_ and various Ni_1−*x*_Co_*x*_PS_3_ samples in the HER, in comparison with the commercial Pt/C catalyst; **b** Tafel plots for the data presented in **a** [72]. Reproduced with permission. Copyright (2017), Royal Society of Chemistry. **c** *J*–*V* curves after *i*R correction for application of FePS_3_, NiPS_3_, and various Ni_1−*x*_Fe_*x*_PS_3_ samples in the HER, in comparison with 20 wt% Pt/C commercial catalyst; **d** Tafel plots for the data presented in **c** [73]. Reproduced with permission. Copyright (2017), American Chemical Society

Beyond the single-metal MPS_3_ compounds, a series of Ni/Co bimetal trithiophosphate nanosheets (Ni_1−*x*_Co_*x*_PS_3_, *x* = 0, 0.03, 0.05, 0.07, and 0.09) were prepared, and their HER performance under alkaline conditions (pH = 14) was evaluated [72]. The few-layered Ni_1−*x*_Co_*x*_PS_3_ nanosheets were obtained by liquid exfoliation. Importantly, after Co doping, the HER performance of these Ni_1−*x*_Co_*x*_PS_3_ nanosheets improved dramatically compared with that of the NiPS_3_ nanosheets. Specifically, Ni_0.95_Co_0.05_PS_3_ exhibited superior HER activity, with an overpotential of 71 mV at a current density of 10 mA cm^−2^ and a Tafel slope of 77 mV dec^−1^ (Fig. 5a, b). This performance was much better than that of the NiPS_3_ and FePS_3_ nanosheets supported by conductive rGO [67, 71]. Electrical conductivity measurements showed that Co doping can dramatically improve the conductivity of NiPS_3_ (by about three orders of magnitude), suggesting changes in the electronic structure of NiPS_3_ after Co doping.

It is well known that Co is quite expensive compared with other transition metals like Mn, Fe, and Ni. Moreover, compared with Co, Fe is earth abundant and more common in high-efficiency hydrogenase. The incorporation of Fe with Ni might also be reasonable for modulating the HER activity of NiPS_3_-based compounds. Therefore, Jin et al. [73] prepared a series of Ni/Fe bimetal trithiophosphate nanosheets (Ni_1−*x*_Fe_*x*_PS_3_ samples, *x* = 0, 0.05, 0.1, 0.15, and 1) as electrocatalysts for the HER. Through DFT calculation of Δ*G*_H_, the authors proposed that H atom adsorption on edge sites is more favorable than on the basal surface. In addition, the Δ*G*_H_ for the S sites in NiPS_3_ is negative, while it is positive for the S and P sites of FePS_3_, implying the existence of optimal intermediates with better HER activity. The results confirmed the speculation that Fe-doped NiPS_3_ can exhibit electrocatalytic HER performance comparable that of the Co-doped counterpart. For the optimized Ni_0.9_Fe_0.1_PS_3_ compound, the overpotential at 10 mA cm^−2^ was 72 mV and the Tafel slope could reach 73 mV dec^−1^ (Fig. 5c, d).

## MPQ_3_ for Electrocatalytic Oxygen Evolution

In the electrochemical water splitting process, the HER is the cathodic half reaction and the OER occurs at the anode. However, the OER process is significantly different from the HER. The general mechanism for the OER process is widely represented as follows [74]:

8 $${\text{In}}\;{\text{alkaline}}\;{\text{solution:}}\;4{\text{OH}}^{ - } \to {\text{O}}_{2} + 2{\text{H}}_{2} {\text{O}} + 4{\text{e}}^{ - }$$

9 $${\text{In}}\;{\text{acidic}}\;{\text{solution:}}\;2{\text{H}}_{2} {\text{O}} \to {\text{O}}_{2} + 4{\text{H}}^{ + } + 4{\text{e}}^{ - }$$

Notably, the OER in alkaline solution can be divided into four steps as follows [75]:

10 $${\text{OH}}^{ - } + {\text{Cat}} \to {\text{Cat}} - {\text{OH}}^{*} + {\text{e}}^{ - }$$

11 $${\text{OH}}^{ - } + {\text{Cat}} - {\text{OH}}^{*} \to {\text{H}}_{2} {\text{O}} + {\text{Cat}} - {\text{O}}^{*} + {\text{e}}^{ - }$$

12 $${\text{OH}}^{ - } + {\text{Cat}} - {\text{O}}^{*} \to {\text{Cat}} - {\text{OOH}}^{*} + {\text{e}}^{ - }$$

13 $${\text{OH}}^{ - } + {\text{Cat}} - {\text{OOH}}^{*} \to {\text{O}}_{2} + {\text{H}}_{2} {\text{O}} + {\text{e}}^{ - } + {\text{Cat}}$$

where OH*, O*, and OOH* are the corresponding free radical species. Based on the calculated Δ*G* calculation, the reaction should overcome the lowest voltage of 1.23 V versus the reversible hydrogen electrode (RHE). In addition, the existence of the above free radical species causes the OER process to always occur at an oxidized surface.

In recent work, Pumera et al. [68] compared the OER activity of a series of bulk MPS_3_ compounds in 1 m KOH solution. The results showed that most of the MPS_3_ compounds exhibited low OER activity, whereas only CoPS_3_ showed higher OER activity than the other analogs. However, the results are dubious because CoPS_3_ can afford a current density of 10 mA cm^−2^ at a potential of 0.84 V versus RHE, which is less than 1.23 V. Recently, our group evaluated the OER performance of bulk and liquid-exfoliated NiPS_3_ nanosheets under alkaline conditions [51]. The exfoliated NiPS_3_ nanosheets provided an overpotential of 301 mV@10 mA cm^−2^, which is about 140 mV lower than that of bulk NiPS_3_. Comparison of the electrochemically active surface area (ECSA) of these two materials indicates that the ECSA of the exfoliated NiPS_3_ nanosheets is about 3.4-fold larger than that of bulk NiPS_3_. Further, the current density of the exfoliated NiPS_3_ nanosheets at 1.55 V is more than fivefold higher than that of bulk NiPS_3_. The nonlinear increase in the current density, along with the ECSA, indicates that the large ECSA of the exfoliated NiPS_3_ nanosheets is not the only reason for the improved OER activity.

A more detailed OER study of exfoliated NiPS_3_ nanosheets was reported by Schuhmann et al. [48]. As mentioned above, the free radical species generated during the OER process might react with the catalyst. As such, surface oxidation cannot be avoided for MPS_3_ materials. By employing a combination of scanning electrochemical microscopy (SECM), in situ Raman spectroscopy, SEM, and XPS measurements, Schuhmann et al. demonstrated that during the OER process, the NiPS_3_ surface is oxidized, resulting in the formation of a NiPS_3_@amorphous NiOOH core–shell heterostructure. According to their studies, the surface oxidation process can be divided into two steps. In the first step, the NiPS_3_ surface is oxidized to Ni(OH)_2_ when immersed in KOH solution. With increasing applied voltage, Ni^2+^ is further oxidized to Ni^3+^, accompanied by the transformation of Ni(OH)_2_ to NiOOH before oxygen evolution. This detailed oxidation mechanism was not provided for previously reported NiS_2_/NiS and NiCoP/C nanoboxes and other MPQ_3_ compounds [51, 68, 76, 77]. Notably, the core–shell heterostructure can provide a current density of 10 mA cm^−2^ with an overpotential of 350 mV, which is lower than that of noble metal oxide catalysts [78, 79]. Furthermore, DFT calculation indicates that the metallic character of the NiPS_3_ nanosheets can provide an electron conduction pathway for efficient transport to surface NiOOH species. The NiOOH species at the surface with a high density of accessible active edges and defect sites can act as oxygen evolution centers. The verification of the relationship between this uniquely formed in situ core–shell heterostructure and the outstanding OER activity provides a useful guideline for understanding the OER mechanism in MPS_3_ materials.

**Fig. 6:**
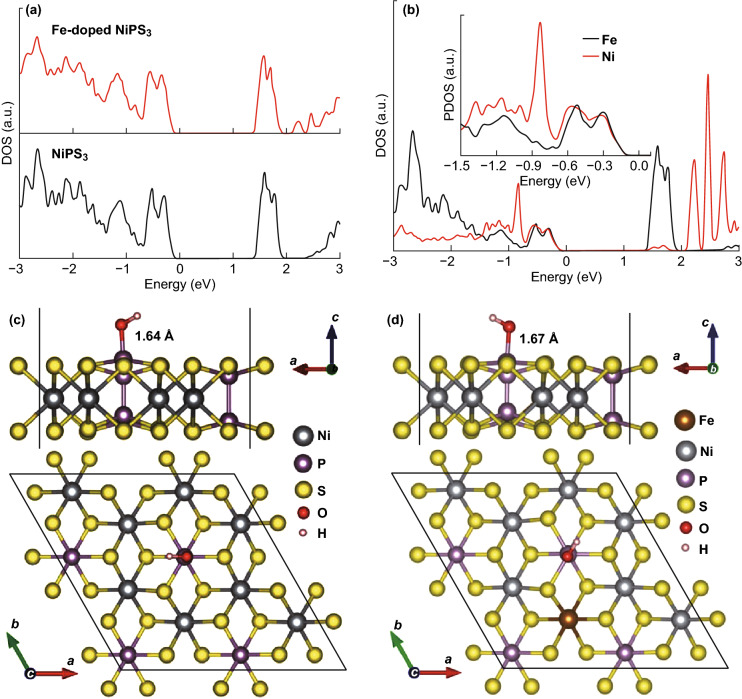
**a** Calculated total DOS for NiPS_3_ and Fe-doped NiPS_3_. **b** Projected DOS for Ni and Fe obtained from DFT + U calculations for Fe-doped NiPS_3_. Optimized structures of **c** NiPS_3_ and **d** Fe-doped NiPS_3_ for calculating the free energies of adsorption of oxygen-containing intermediates on their surfaces by DFT calculations. The inset in **b** is the enlarged projected DOS near the Fermi level [80]. Reproduced with permission. Copyright (2018), Elsevier Ltd.

Studies on the OER performance of Ni/Fe bimetal trithiophosphates have also been documented [73, 80]. As reported by Jin and co-workers [73], Ni/Fe bimetal trithiophosphate nanosheets (Ni_1−*x*_Fe_*x*_PS_3_ samples, *x* = 0, 0.05, 0.1, 0.15, 1) could be synthesized by a traditional CVT method, followed by liquid exfoliation. The resulting FePS_3_ was almost inactive for OER catalysis, whereas the Ni_1−*x*_Fe_*x*_PS_3_ nanosheets showed much better OER performance than the undoped NiPS_3_. Ni_0.9_Fe_0.1_PS_3_ exhibited the best OER activity among all the Ni_1−*x*_Fe_*x*_PS_3_ samples, providing a current density of up to 20 mA cm^−2^ at an overpotential of 329 mV. In comparison, our group developed a novel solid-state transformation (SST) process for the mass production of MPQ_3_ nanosheets [80]. Using this method, Fe-doped NiPS_3_ nanosheets (Fe = 5.93% in atomic percentage) with a smaller lateral size (~ 100 nm) than the exfoliated congeners (several micrometers) could be prepared. Interestingly, the OER performance of the Fe-doped NiPS_3_ nanosheets was much better than that of the NiPS_3_ and Fe-doped Ni(OH)_2_ nanosheets, as well as that of the exfoliated Ni_0.9_Fe_0.1_PS_3_ nanosheets. The optimized sample showed an overpotential of 256 mV to reach a current density of 30 mA cm^−2^. The Tafel slope of this Fe-doped NiPS_3_ nanosheet (46 mV dec^−1^) was also better than that of IrO_2_ (56 mV dec^−1^) and RuO_2_ (86 mV dec^−1^). As demonstrated by the ECSA tests, electrochemical impedance spectroscopy (EIS), and DFT calculation, the superior electrocatalytic activity of the Fe-doped NiPS_3_ nanosheets might be ascribed to the Fe-doping. DFT calculation revealed that doping with Fe could improve the electronic conductivity and significantly weaken the interaction between NiPS_3_ and the oxygen-containing intermediates (Fig. 6). Combined with all of the above-mentioned theoretical studies related to electrocatalytic water splitting, metal doping in MPQ_3_ materials is a prospectively efficient way to achieve intermediate binding energy of hydrogen/oxygen species (and, therefore, improved catalytic performance). In this regard, the integration of metals that fall on the opposite slopes of the volcano plots should be reasonable for the design of multicomponent MPQ_3_ catalysts [7, 81].

Furthermore, we also designed a series of novel 0D–2D nanohybrids composed of a Ni_1−*x*_Fe_*x*_PS_3_ (*x* = 0.1, 0.2, 0.3, 0.4) nanomosaic decorated on the surface of Ti_3_C_2_T_*x*_*@*MXene [82]. Here, nanomosaic refers to Ni_1−*x*_Fe_*x*_PS_3_ nanoparticles with irregular shapes that are dozens of nanometers in lateral size, which covered the surface of MXene to form a mosaiclike structure. Such a heterostructure integrating the Ni_1−*x*_Fe_*x*_PS_3_ nanomosaic and conductive Ti_3_C_2_T_*x*_ MXene can combine the advantages of each constituent to significantly enhance the conductivity and active sites. The resulting nanohybrids exhibited improved HER and OER performance relative to the individual materials. Among all the tested samples, the Ni_0.7_Fe_0.3_PS_3_@MXene nanohybrid, as an OER catalyst, showed excellent catalytic performance (282 mV@10 mA cm^−2^) and a Tafel slope of 36.5 mV dec^−1^. Further, the Ni_0.9_Fe_0.1_PS_3_@MXene nanohybrid showed optimized HER performance in alkaline solution (with an overpotential of 196 mV). Due to their efficacy for the electrocatalytic HER and OER, overall water splitting could be achieved with the nanohybrid-based catalysts. The Ni_0.7_Fe_0.3_PS_3_@MXene||Ni_0.9_Fe_0.1_PS_3_@MXene couple showed a low onset potential of 1.42 V and a potential of only 1.65 V to reach a current density of 10 mA cm^−2^.

Finally, a comparison of all the MPQ_3_ compounds with known electrochemical catalytic properties is presented in Table 1. The conditions used to evaluate each compound, including the supporting electrode, the electrolyte, and the overpotential, as well as the corresponding Tafel slope and corresponding references, are also given. As summarized in Table 1, without metal doping, the NiPS_3_ nanostructures generally show an overpotential of about 300 mV under acidic conditions for the HER. Under alkaline conditions, the value was further increased to about 400 mV. The performance of NiPS_3_ is worse than that of most of the alloys, metal carbide, metal sulfide, and metal phosphide catalysts [3]. However, when Fe or Co is introduced as a dopant, the overpotential can be dramatically decreased to around 70 mV under alkaline conditions. This value is less than that of most of the alloy-based and metal carbide/nitride catalysts, which suggests better HER performance. The same phenomenon was observed in the OER studies. For the OER under alkaline conditions, NiPS_3_ shows an overpotential of 350–450 mV, which is only comparable with that of some metal oxides and phosphides [3, 83, 84]. Nevertheless, for Fe-doped-NiPS_3_, the overpotential could reach 256 mV@30 mA cm^−2^, which is better than that of the metal oxides, metal layered double hydroxides, and some metal phosphides [83–86].

**Table 1:** Comparison of electrochemical catalytic properties of reported MPQ_3_ compounds

Sample	Support	Electrolyte	*η*_HER_^a^ (mV)	TS_HER_^b^ (mV dec^−1^)	*η*_OER_^c^ (mV)	TS_OER_^d^ (mV dec^−1^)	References
Few-layered NiPS_3_	GCE^*e*^	0.5 m H_2_SO_4_	297	69			[67]
		3.5 wt% of NaCl	816	159			
		1 m KOH	398	54			
rGO–few-layered NiPS_3_	GCE	0.5 m H_2_SO_4_	178	55			
		3.5 wt% of NaCl	543	94			
		1 m KOH	281	48			
Few-layered FePS_3_	GCE	0.5 m H_2_SO_4_	211 ± 3	42			[71]
		3.5 wt% of NaCl	673 ± 4				
		1 m KOH	337 ± 4				
rGO–few-layered FePS_3_	GCE	0.5 m H_2_SO_4_	108 ± 2	54			
		3.5 wt% of NaCl	467 ± 3				
		1 m KOH	192 ± 2				
Ni_0.97_Co_0.03_PS_3_	RDE^*f*^	1 m KOH	112	103			[72]
Ni_0.95_Co_0.05_PS_3_			71	77			
Ni_0.93_Co_0.07_PS_3_			105	110			
Ni_0.91_Co_0.09_PS_3_			145	113			
NiPS_3_	GCE	1 m KOH	193	221	437@20 mA cm^−2^	73	[73]
Ni_0.95_Fe_0.05_PS_3_			130	114	359@20 mA cm^−2^	87	
Ni_0.9_Fe_0.1_PS_3_			72	73	329@20 mA cm^−2^	69	
Ni_0.85_Fe_0.15_PS_3_			152	187	356@20 mA cm^−2^	101	
FePS_3_				109		419	
NiPS_3_	GCE	1 m KOH			301	43	[51]
NiPS_3_@NiOOH	RDE	1 m KOH			350	80	[48]
Ni_0.9407_Fe_0.0593_PS_3_	GCE	1 m KOH			256@30 mA cm^−2^	46	[80]
NiPS_3_@MXene	GCE	1 m KOH	364	167	406	108.8	[82]
Ni_0.9_Fe_0.1_PS_3_@MXene			196	114	312	48.9	
Ni_0.8_Fe_0.2_PS_3_@MXene			297	137	306	40.9	
Ni_0.7_Fe_0.3_PS_3_@MXene			359	140	282	36.5	
Ni_0.6_Fe_0.4_PS_3_@MXene			475	173	305	39.7	

^a^HER overpotential of at the current density of − 10 mA cm^−2^

^b^Tafel slope of HER

^c^OER overpotential of at the current density of 10 mA cm^−2^

^d^Tafel slope of OER

^e^*GCE* glassy carbon electrode

^f^*RDE* rotating disk electrode

## Summary and Outlook

In this minireview, recent advances in the study of metal trichalcogenidophosphates for photo- and electrocatalytic water splitting were briefly summarized. Theoretical calculations have revealed the possibility of adjusting the electronic structure of MPQ_3_-based materials through composition tuning, doping, and heterostructure interface conjunction. The success of MPQ_3_ catalysts with various compositions and structures for overall water splitting has confirmed their promising practical application. There is no doubt that MPQ_3_-based materials are good candidates as high-performance photo- or electrocatalytic water splitting catalysts. However, studies on the catalytic properties of MPQ_3_ are still in the early stage, and there are still many challenges related to MPQ_3_ that must be overcome, such as preventing anodic corrosion during the photocatalytic water splitting process, proper selection of doping metals, probing the active site, and exploring the catalytic mechanism during the HER and OER processes.

In the near future, the most urgent task related to MPQ_3_ catalysts is understanding the relationships among the composition, electronic structure, and adsorption free energies of the reactive intermediates on the MPQ_3_ surface. Systematic understanding of doping metal selection is needed. Moreover, the construction of MPQ_3_-based heterostructures is another important undertaking for achieving high-performance water splitting catalysts. In this regard, more effort should be devoted to control of the carrier separation and migration in MPQ_3_-based heterostructures by carefully selecting the co-catalysts.
